# Autophagy Is a Promoter for Aerobic Exercise Performance during High Altitude Training

**DOI:** 10.1155/2018/3617508

**Published:** 2018-04-05

**Authors:** Ying Zhang, Ning Chen

**Affiliations:** ^1^Graduate School, Wuhan Sports University, Wuhan 430079, China; ^2^College of Sports, Hubei University of Science and Technology, Xianning 437100, China; ^3^Tianjiu Research and Development Center for Exercise Nutrition and Foods, Hubei Key Laboratory of Sport Training and Monitoring, College of Health Science, Wuhan Sports University, Wuhan 430079, China

## Abstract

High altitude training is one of the effective strategies for improving aerobic exercise performance at sea level via altitude acclimatization, thereby improving oxygen transport and/or utilization. But its underlying molecular mechanisms on physiological functions and exercise performance of athletes are still vague. More recent evidence suggests that the recycling of cellular components by autophagy is an important process of the body involved in the adaptive responses to exercise. Whether high altitude training can activate autophagy or whether high altitude training can improve exercise performance through exercise-induced autophagy is still unclear. In this narrative review article, we will summarize current research advances in the improvement of exercise performance through high altitude training and its reasonable molecular mechanisms associated with autophagy, which will provide a new field to explore the molecular mechanisms of adaptive response to high altitude training.

## 1. Background

High altitude training is one of the effective strategies for the improvement of exercise performance for many exercise events. Since it has been proposed in the 1960s, high altitude training has been extensively explored, but it is still debated among exercise physiologists all over the world [1–3]. Currently, high altitude training has gained tremendous attention and extensive investigation as the hot topic for improving exercise performance of athletes. The dual stimulation from hypoxia and exercise training can induce high altitude acclimatization, thereby increasing respiratory frequency [4], accelerating heart rate [5], elevating hemoglobin level and red cell volume [6], improving blood volume [7], promoting blood flow rate, enhancing capillary density [8], and reducing blood lactate concentration [9], which can further improve the function of cardiovascular system, local blood supply, lactic acid tolerance capacity, and maximal oxygen consumption (VO_2max_) of the athlete [10]. Its underlying molecular mechanisms on physiological functions and exercise performance of athletes are still vague. Similarly, autophagy has been confirmed to be involved in a series of physiological and pathological processes. Autophagy is a cellular self-consumption process characterized by the sequestration of bulk cytoplasm, long-lived proteins, and damaged cellular organelles in double membrane autophagosomes that are delivered to lysosomes for degradation [11], which process can be activated under the situations in response to starvation [12], oxidative stress [13], hypoxic stimulus [14], or organelles, and nonfunctional protein accumulation [15]. The organism during exercise has a sharp increase in the consumption of energy and oxygen, thus resulting in cellular deprivation of nutrition and oxygen, and increased reactive oxygen species (ROS) generation [16] and calcium [17], and correspondingly inducing autophagy to adapt the unflavored cellular environment. Meanwhile, exercise-induced autophagy can be observed in multiple tissues and organs including skeletal muscle, cardiac muscle, liver, pancreas, adipose tissue, and cerebral cortex [18–21]. Previous studies have shown that exercise training can enhance autophagy to maintain normal physiological activities of skeletal muscle [22], inhibit apoptosis caused by myocardial infarction, reduce myocardial cell damage, and improve cardiovascular function [23]. But physical inactivity or excessive exercise may cause excessive activation of autophagy or apoptosis, thus resulting in muscle atrophy [24] and myodynamia [25].

On the other hand, whether high altitude training can activate autophagy or whether high altitude training can improve exercise performance through an autophagy-dependent or autophagy-independent manner is still unclear. In this article, we attempt to summarize current research advances of high altitude training in the improvement of exercise performance to explore its corresponding molecular mechanisms or signal pathways associated with autophagy.

## 2. Autophagy

In 1963, Christian de Duve first proposed the concept of autophagy. The word of autophagy is originally derived from Greek. “Auto” means self, and “phagy” means to eat, suggesting that autophagy is the process for digesting cell's own materials [26, 27]. Autophagy presents in all types of eukaryotic cells with the involvement of normal or abnormal development of cells so that it is also associated with a series of diseases [28–31]. Autophagy is an important process for the degradation of endogenous substrates such as long-lived proteins and damaged cellular organelles in cells in the presence of lysosomes. Autophagy can stabilize intracellular environment through modulating cell survival, cell self-renewal, and vitality [32].

### 2.1. Classification of Autophagy

Usually, autophagy can be divided into three forms including macroautophagy, microautophagy, and chaperone-mediated autophagy [33]. Among these three forms of autophagy, macroautophagy is the most widely studied. The following mentioned autophagy refers to macroautophagy. According to its selectivity of degradation, autophagy can be divided into nonselective autophagy and selective autophagy [34, 35]. The nonselective autophagy refers to nonselectively degrading cytoplasmic components, and the selective autophagy refers to selectively degrading cellular organelles or components [36], which includes mitochondrial autophagy (mitophagy), endoplasmic reticulum autophagy (ER-phagy), peroxisomal autophagy (pexophagy), and lipophagy [37].

### 2.2. The Process and Regulatory Molecular Mechanisms of Autophagy

The autophagic process, an independent double membrane structure-phagocytic vacuole, is initiated and extended for engulfing aged or denatured proteins and DNAs as well as dysfunctional organelles for the degradation once the formed autophagosomes are coupled with lysosomes [27, 38]. The regulation of autophagy mainly depends on the coordination and cooperation between the system and signal pathway. Autophagy is mainly regulated autophagy-related genes (*Atg*s). So far, about 40 *Atg*s have been identified during the regulatory processes; most of which are also highly conservative in eukaryotes [38].

#### 2.2.1. Atg1/ULK1 Kinase Complex Induces the Initiation of Autophagy

Atg1/ULK1 kinase complex plays an important role in autophagy initiation. In mammalian cells, Atg1 homologue that initially found to be able to trigger autophagy [39] is Unc-51-like autophagy activating kinase 1 and 2 (ULK1 and ULK2) [40, 41]. In the absence of glucose or energy, ULK1 activity can be regulated by adenosine monophosphate-activated protein kinase (AMPK). AMPK can result in the direct activation of ULK1 by phosphorylating multiple serine sites in the central region of ULK1 [42]. AMPK also can activate ULK1 indirectly by inhibiting mammalian target of rapamycin complex 1 (mTORC1) through phosphorylating raptor subunit of mTORC1 [43], thereby resulting in the phosphorylation of downstream Atg13, Beclin1, and VPS34 substrates [44], and then inducing the occurrence of autophagy. The regulation of signal pathway associated with autophagy can be divided into mammalian target of rapamycin- (mTOR-) dependent signal pathway and mTOR-independent signal pathway [45]. Under the condition of hypoxia and starvation, the occurrence of autophagy is mainly regulated by mTOR signal pathway [44].

#### 2.2.2. PI3K Kinase Complex Participates in the Expansion and Maturation of Autophagic Vacuoles

Class III phosphoinositide 3-kinase (PI3K) plays the most important role in autophagy in mammals. It can phosphorylate phosphatidylinositol to produce phosphatidylinositol 3-phosphate (PI3P) [46] and then participate in the formation and maturation of autophagic vacuoles. More than 30 Atg proteins are assembled into a preautophagosomal structure (PAS) on the vesicular membrane [47]. Beclin1/(Atg6) is used as the scaffolding protein for class III PI3K complex [48], which can combine with Atg9, Atg14L, and UVRAG to form a PI3K core complex [49] via the combination of VPS34-p150 (VPS15 orthologous), thereby triggering autophagy.

With the support of the ubiquitin coupling system, Atg10, Atg7, Atg3, Atg8/LC3, Atg4, and Atg12-Atg5-Atg16L1 are eventually recruited to PAS and then involved in vesicular elongation and autophagosome maturation. LC3-II is a surface marker of autophagic vacuoles, and the amount of LC3-II can indirectly reflect the level of autophagy in cells [50]. LC3-I in cytoplasm combined with phosphatidylethanolamine (PE) through a series of catalytic Atg7 and Atg3 to form membrane-bound LC3-II that is localized in autophagy precursor and autophagosome membranes. Once autophagosomes fuse with lysosomes, LC3-II can be degraded by hydrolytic enzymes in lysosomes [51]. Therefore, LC3-II or LC3-II/LC3-I ratio is commonly used as the molecular marker for evaluating the induction of autophagy [52].

#### 2.2.3. p62/SQSTM1 Is Involved in the Degradation of Autophagolysosomes

Autophagosomes are fused with lysosomes to form autophagolysosomes through acidification. Autophagic adaptor p62/SQSTM1 executes specific identification, separation, and transport [37] of p62 or its substrate degradation, thus releasing nutrients and ATP for cell recycling [53]. In addition, p62 also has a negative correlation with autophagy activity, which reflects lysosome activity of autophagolysosomes and autophagic flux for evaluating the functional status of autophagy [54].

## 3. High Altitude Training Improves the Function of Skeletal Muscle

Over the decades, endurance athletes try to improve exercise performance through high altitude training. High altitude training can result in the increase of VO_2max_, but the exercise performance is not completely associated with VO_2max_. High altitude training can also result in other changes of nonblood factors [10], such as energy saving, lactic acid threshold, and oxygen utilization of muscle. Upon the stimuli from hypoxia and exercise, the body can produce a variety of adaptive responses such as increased muscle mass and capillary number in skeletal muscle and increased ratio of capillary and fibers. Previous study has demonstrated that high intensity training under hypoxic environment can promote the mRNA expression of vascular endothelial growth factor (VEGF) in skeletal muscle, thus improving oxygen transportation and intake in muscle tissues [55, 56]. In addition, hypoxic training can also enlarge the cross-sectional area (CSA) of skeletal muscle [57]. The body compositions after a 3-week high altitude exposure have shown that training in high altitude presents increased muscle mass and decreased body mass at the same time [58]. Exercise is also an important factor that induces protein synthesis in muscle tissue and muscle hypertrophy via activating Akt-mTOR-p70S6K signal axis. Treadmill training can attenuate the expression of MuRF1, atrogin-1, and myostatin (Mstn) and recover protein kinase B (Akt or PKB) and p70S6 kinase activity as well as forkhead box O3 (FoxO3) phosphorylation during the impact of cisplatin, thereby mitigating cisplatin-induced muscle atrophy [59]. Although the increase in CSA and strength endurance or capacity of muscle is observed in the hypoxia group, there are no indications that hypoxic training is superior to normoxic training [60]. Furthermore, the study has demonstrated that exercise training can synergize hypoxic stress stimuli and reduce blood flow in skeletal muscle, correspondingly resulting in the decreased protein synthesis and the attenuated shrinking and loss of skeletal muscle fibers [61]. The decrease in muscle fibers results in the enhanced oxygen diffusion into muscle cell and reduced protein synthesis and energy consumption, which demonstrates the energetic challenge in skeletal muscle through metabolic optimization during hypoxic training [62].

Protein degradation in skeletal muscle is mainly controlled by two proteolytic systems, namely ubiquitin proteasome system (UPS) and autophagy-lysosome system (ALS). Several studies have shown that autophagy is required for the control of skeletal muscle mass under catabolic conditions and plays an important role in maintaining the homeostasis and integrity of skeletal muscle [63], especially the autophagy at the appropriate level. Previous studies have proved the importance of autophagy in skeletal muscle of Atg7 gene knockout rats, and autophagy inhibition can lead to muscle atrophy and myopathy [64]. The functional status or level of autophagy is the determinant for the health of skeletal muscle. High-level autophagy can cause the decrease in the quality of skeletal muscle; similarly, too low-level autophagy can result in the excessive degradation of skeletal muscle or myasthenia [65]. The decreased LC3 level is observed in aged muscle, which illustrates that sarcopenia is highly correlated with the deficient or dysfunctional autophagy [18, 66]. Thus, the reduction in the adaptive plasticity of aged muscle is associated with the decrease in disuse-induced autophagy. These data indicate that the expression of autophagy-related proteins and their localization to mitochondria are not decreased in aged muscle; however, the induction of autophagy in response to disuse, along with downstream events such as lysosome function, is impaired. This may contribute to an accumulation of dysfunctional mitochondria in aged muscle [67]. The increased expression of Beclin1 and LC3B proteins is observed in cachectic cancer patients, suggesting autophagy induction in cancer-induced muscle wasting [68]. The aggregation of ubiquitin and p62/SQSTM1 proteins has also been observed in skeletal muscle of the patients with myopathy- or autophagy-specific gene knockout rats and the mice with sarcopenia [66, 69]. Moreover, p62/SQSTM1-positive fibers are significantly smaller than p62/SQSTM1-negative fibers in mice [66]. Autophagy defects also can result in abnormal glucose metabolism and decreased exercise capacity of mice [70].

The adaptation of skeletal muscle to exercise and the improvement of exercise performance are highly correlated with the activation of autophagy [22]. There is difference in basal autophagic protein expression and autophagic flux between oxidative and glycolytic muscle. Under the basal autophagy, slow-twitch muscle fibers are obviously higher than hybrid fibers and fast-twitch muscle fibers [22]. Compared with oxidative muscle, specific autophagic markers (LC3-I, LC3-II, and SQSTM1) are basally lower, but basal autophagic flux is higher in glycolytic muscle [63].

The autophagy in skeletal muscle response to endurance exercise is first described in 1984. The dramatic high-intensity treadmill training of mice has shown that the strongest autophagy can be observed within 2–7 days after exercise. Under the observation by a microscope, different degradation stages of mitochondria are observed in the manner of autophagic vacuole, which can be speculated that autophagy may be “obstacles” for the generation of new muscle fibers after exercise [71]. The 8-week eccentric endurance training can improve the expression of LC3, Atg7, and Beclin1 in skeletal muscle of aging mice, indicating the activation of autophagy [72]. The upregulation in autophagy-related genes has also been documented that the transcript levels of autophagy-related genes such as *Atg4b*, Gabarap1, LC3, and BNIP3 are enhanced after ultra-endurance exercise, indicating that autophagy can be activated in human muscle in response to ultra endurance exercise [73]. On the other hand, acute resistance training and high-resistance contraction can significantly improve the activity of mammalian VPS34 (mVPS34) that is responsible for the regulation of autophagy through Beclin1-VPS34 and prolong the activity of mTOR signal pathway [74, 75]. When skeletal muscle is subjected to chronic resistance contraction, the autophagy level in skeletal muscle of aging rats can be enhanced by activated insulin-like growth factor 1 (IGF-1) and its receptor, Akt/mTOR and Akt/FoxO3 signal pathways, and reduced apoptosis, thus completing the improvement of strength and quality of skeletal muscle [76]. Previous studies have shown that increased basal autophagy is required for exercise-induced metabolic adaptations [22]. Through activating autophagy, long-term exercise-induced AMPK and Sestrin interaction may contribute to the beneficial metabolic effects, thereby indicating that exercise-induced autophagy can promote glucose uptake in skeletal muscle [77].

However, excessive exercise training can lead to excessive autophagy and induce atrophy-related gene expression or excessive protein degradation, injury, or loss of skeletal muscle, which is observed in *Vastus lateralis* from male subjects with 20 km distance running [78]. In this study, the significantly increased protein expression of Atg12 and LC3-II and MuRF1 is observed. These factors are in charge of the modulation of ALS and UPS pathways for accelerating protein degradation and inducing the atrophy of skeletal muscle. Altogether, there is a robust evidence that autophagy in skeletal muscle plays an important role in maintaining the quality of skeletal muscle. The regulation of protein degradation by autophagy is of equal importance as protein synthesis, so that skeletal muscle can obtain the beneficial metabolic effects following endurance exercise training. However, whether the improvement of aerobic capacity under hypoxic environment is associated with the enhancement of energy metabolism induced by activated autophagy following hypoxic exercise is highly desired for further exploration.

## 4. High Altitude Training Improves Cardiac Function

Cardiac function is the limiting factor that affects the endurance level of athletes, and high altitude training is a common method to improve the endurance exercise capacity of athletes [79]. In 1960, Hurtado's report has documented that people who lived at high altitude are more likely to enhance myocardial tolerance to ischemic and hypoxia [1]. However, people who lived in the plateau have more coronary arteries than those who lived in the plains [80]. For many years, researches have described that intermittent hypoxic adaptation and long-term high-altitude hypoxic adaptation have an obvious protective effect on the heart. After hypoxic training, significant increase in cardiac output and heart-stroke index is observed, but the heart rate change is not obvious, suggesting that low-oxygen training can improve cardiovascular function and the ability of the heart to fight ischemia and hypoxia. The change of cardiac function in rats subjected to hypoxic training demonstrates that low oxygen can result in pulmonary hypertension, hypertrophy of the right heart, and temporarily decreased weight of the left ventricular [81]. In addition, hypoxic stimulus can upregulate mRNA expression level of HIF-1 subunits, activate VEGF gene transcription, enhance the stability of VEGF, promote angiogenesis, and increase capillary density, which contributes to the supply of oxygen and nutrients in tissues [82]. In normoxia, moderate-intensity endurance training can result in obvious hyperplasia and significant increase in volume of rat myocardial cell mitochondria, as well as improved mitochondrial function [83]. A series of adaptive responses are produced in the morphology, structure, metabolism, and function of the heart [84].

In chronic hypoxia condition, myocardial cells adapt to chronic hypoxia stress such as energy stress, oxidative stress, and imbalanced calcium ion concentrations by adjusting energy metabolism, oxygen sensitivity, and calcium balance [85], or through increasing mitochondrial biogenesis, altering the activity of mitochondrial respiratory chain-linked enzymes, enhancing the glycolysis to increase myocardial cell ATP production and to meet energy demands of the heart under the condition of chronic hypoxia [86]. In hypoxia, calcium/calmodulin-dependent-like protein kinase (CaMMK) is activated by ROS accumulation and then upregulates the expression of mitochondrial biosynthesis regulator, peroxisome proliferator-activated receptor gamma (PPAR-γ) coactivator 1alpha (PGC-1*α)*, to increase the number of mitochondria [87]. Intermittent aerobic exercise (80–90% maximum oxygen consumption rate) can significantly improve protein expression levels of PPAR-*γ*, PGC-1*α*, and mitochondrial transcription factor in cardiomyocytes postmyocardial infarction [88], illustrating that exercise training can improve mitochondrial biosynthesis postmyocardial infarction. Intermittent aerobic exercise training can reverse the reduced expression of mitofusin 2 (MFN2) and optic atrophy 1 (OPA1) and inhibit the increased expression of dynamin-related protein 1 (DRP1) caused by myocardial infarction [89], which suggests that exercise training can promote mitochondrial membrane fusion of cardiomyocytes, improve mitochondrial energy metabolism, increase resistance capacity to oxidative stress of cardiomyocyte postmyocardial infarction [89].

On the other hand, under the environment with exercise, anoxic or aerobic respiration, and reduced ATP production, AMPK as energy-sensitive protein can be activated mainly through the increased AMP/ATP ratio so as to increase glucose transporter and ATP production [90–92]. Phosphor-AMPK can modulate the metabolism by regulating the intake and oxidation of fatty acids [93]. Activated AMPK increases the amount of glucose transporter 4 (GLUT4) in the membrane of cardiomyocytes [94]. The myocardial ischemia can induce autophagy to maintain ATP level and promote cell survival. AMPK is activated when ATP is deficient or AMP is over accumulated, and AMPK is the key protein involved in the regulation of myocardial autophagy [95]. Previous study has confirmed that the shortage of glucose and myocardial ATP level can induce autophagy, while the ATP level reveals further fall in the presence of autophagy inhibitor 3-methyladenine (3-MA), which illustrates that autophagy can improve the survival of myocardial cells by maintaining ATP at the appropriate level in myocardial ischemia [96]. AMPK induces autophagy through inhibiting mTORC1 by phosphorylating tuberous sclerosis 1/2 (TSC1/2) and mTOR; on the other hand, AMPK can phosphorylate ULK1 to activate BECN1-VPS34-VPS15 complex [49, 97]. Another study has also demonstrated that AMPK has the effect on reducing the risk of myocardial apoptosis in cardiomyocytes during hypoxia/reoxygenation injury [98].

Exercise training at the appropriate intensity can induce autophagy to degrade metabolic wastes so as to maintain the steady state of the cell [19]. Aerobic exercise also can induce autophagy to protect myocardial cells [99, 100]. According to the previous study, LC3-II/LC3-I ratio in mouse myocardium is more significantly higher in the exercise group when compared with the nonexercise group, suggesting that exercise training can reduce myocardial infarction during myocardial cell injury in mice, enhance cardiovascular function by improving autophagy, and promote the degradation of damaged proteins [23]. Autophagy is activated to remove the damaged mitochondria and reduce the release of cytochrome C (Cyt C), thereby delaying or inhibiting apoptosis, which plays the protective role in myocardial cells [101]. Autophagy provides the material basis for myocardial development and survival through degrading denatured proteins and generating amino acids in myocardial cells with normal and moderate stress. The myocardial ischemia can induce autophagy to maintain ATP level in cells, thus maintaining myocardial energy metabolism and function, as well as promoting myocardial cell survival. Myocardium is rich in mitochondria, while adverse environment can lead to mitochondrial damage, thus releasing apoptosis factors and inducing apoptosis [70]. Autophagy can degrade and recycle organelle components to selectively remove damaged mitochondria. High altitude training under dual stimuli of hypoxia and exercise training induces the autophagy of cardiomyocytes to remove the aged mitochondria during oxidative stress or oxidative damage, so as to ensure sufficient number of healthy myocardial mitochondria for the maintenance of energy conversion at high efficiency, which could be the reasonable mechanism for increasing aerobic exercise capacity by high altitude training.

## 5. High Altitude Training Induces Mitophagy to Improve Energy Metabolism

Mitochondria are sensitive to the change in internal and external environments of cells, thus regulating the metabolism of the cells as an important hub for the control of cell survival and death signal pathways. Mitochondria are the most sensitive organelles to hypoxia. Its response to hypoxia is mainly due to oxidative stress and energy metabolism disorders to adjust the adaptive change in the morphology and function of mitochondria [102]. During high altitude training, the body can produce a large number of ROS, and excessive ROS can easily cause oxidative damage of tissues [103]. At a normal physiological state, 95% of ROS in cells are derived from mitochondria [104]. ROS is the by-product during the respiration process of mitochondria and can be neutralized by antioxidant systems in the body. Mitochondria are not only the major places to produce endogenous free radicals, but also the target for the attack from free radicals [105]. Excessive ROS can produce extensive damage to mitochondria, decrease the membrane potential and the opening of mitochondrial permeability transition (MPT) pore, and release proapoptosis and necrosis factors [106].

In 2005, a study has demonstrated the mitochondrial autophagy due to the decrease of mitochondrial membrane potential and the opening of MPT pore, in which the concept of mitochondrial autophagy (mitophagy) is formally put forward [107]. Mitophagy often occurs in dysfunctional mitochondria following damage or stress. As a defense mechanism for the removal of impaired mitochondria and excessive production of ROS, mitophagy ensures the stability of mitochondrial function in cells [30]. Under the situation of defective mitophagy, the overproduction of ROS will induce apoptosis [108]. In order to maintain the homeostasis of cells, the removal of impaired or unnecessary mitochondria for maintaining the balance of the quality and quantity of mitochondria is highly required [109]. When cells are in a harsh environment, too many mitochondria will be aggravated [110]. Mitophagy can promote the turnover of mitochondria and prevent the accumulation of impaired mitochondria [111]. The decreased mitochondrial membrane potential may lead to mitophagy, which requires the involvement of Parkin and phosphatase and tensin homolog- (PTEN-) induced putative kinase 1 (Pink1) [112, 113]. Under the condition with starvation, rapid degradation of mitochondria from cultured liver cells can be observed, and the inhibitor of MPT pore cyclosporin A (CsA) can inhibit membrane potential to reduce and suppress mitophagy at the same time [114]. The membrane potential of some progeny of mitochondria during division is too low and functional disorder, so that they will be degraded by mitophagy in priority.

Long-term exposure in the plateau environment can result in mitochondrial autophagy and reduced total mitochondrial volume or density in skeletal muscle [115]. Oxidative stress can be stimulated by hypoxia, and ischemia/reperfusion, thus upregulating Beclin1 and inducing autophagy [116, 117]. Beclin1 combined with PI3K can adjust the localization of ATG proteins in autophagy precursor structure [118]. The expression of BNIP3 induced by hypoxia-inducible factor 1 (HIF-1) plays an important role in the constitutive expression of Beclin1 and Atg5, indicating that hypoxia can induce mitochondrial autophagy in cells [119]. HIF-1 can modulate the interaction between B-cell lymphoma 2 (Bcl-2) and BNIP3, which leads to the selective autophagy of mitochondria or inhibits mTOR for inducing autophagy [120]. HIF-1a as the oxygen balance-regulating transcription factor is recognized as the “master regulator” of hypoxia signaling in cells [115]. Under hypoxic environment, HIF-1 is activated, and abundant accumulation of HIF-1a subunit can initiate the transcription of a variety of low-oxygen reaction genes and induce the expression of target genes [121], thus correspondingly initiating a wide range of adaptation to maintain oxygen delivery. The NIX-dependent (also called BINP3L-dependent) decrease of mitochondrial membrane potential is important during the process of mitophagy [122]. Recent studies have shown that the phosphorylation of LC3-interacting region (LIR) in Bnip3 can promote the interaction between Bnip3 and LC3B to induce mitophagy, thereby resulting in the clearance of damaged mitochondria, and reduce mitochondrial injury in myocardial cells [123]. In addition, previous studies have also demonstrated that the increase of erythropoietin (EPO) is an adaptive response to hypoxia caused by HIF-1. HIF-1 has been confirmed to modulate the expression of genes responsible for iron absorption and transport and hemoglobin synthesis [115]. It is reported that NIX plays a key role in the regulation of erythroid maturation through mitophagy. NIX^−/−^ mice are characterized by developing anemia with reduced matured erythrocytes and compensatory expansion of erythroid precursors. Erythrocytes in the peripheral blood of NIX^−/−^ mice show mitochondrial retention and reduced lifespan [122]. Previous studies have also shown that reticulocytes become matured red blood cells through mitophagy to remove dysfunctional mitochondria, and BNIP3L of mitochondria-mediated interaction process is correlated with mitophagy [124].

Exercise training can promote the biogenesis of mitochondria; at the same time, it also can remove the aged or damaged mitochondria by mitophagy to ensure sufficient number of healthy mitochondria for maintaining high efficiency of energy metabolism [125]. Therefore, moderate exercise training is an effective way against the injury caused by high-altitude hypoxic exposure [126]. Hypoxia can induce mitochondrial autophagy. Exercise also can promote mitophagy. Exercise training under low-oxygen condition can cause mitochondrial stress response to hypoxic stimuli. If excessive ROS induced by high altitude training can cause mitophagy to maintain cellular functions in hypoxic environment and the signal pathways of mitophagy induced by hypoxic training should be further explored. In addition, although autophagy or microRNA-mediated autophagy is involved in regulating the removal of organelles to promote the adaptation to exercise, and functional prevention, recovery, and improvement through exercise intervention [31, 127, 128], the underlying molecular mechanisms remain to be explored. Similarly, whether the increase of erythrocytes caused by high altitude training is correlated with mitophagy and erythrocyte maturation is also highly necessary for further investigation.

## 6. The Prospects of High Altitude Training

**Figure 1:**
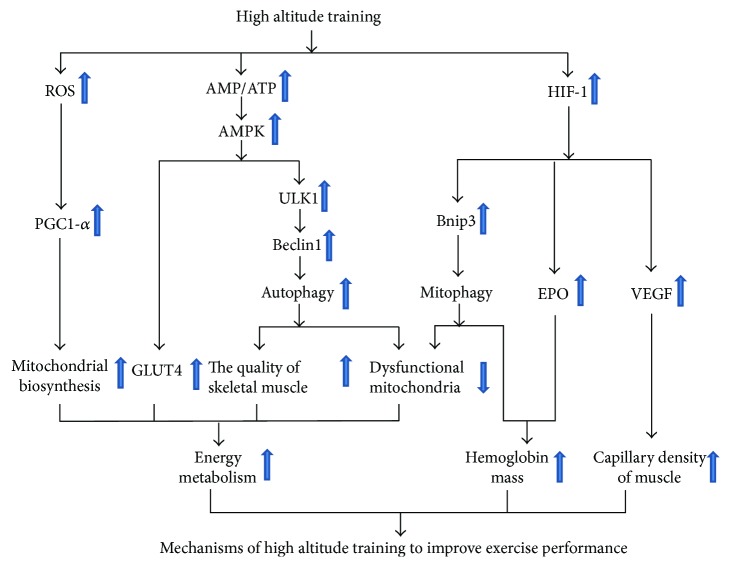
The comprehensive underlying mechanisms of high altitude training to improve exercise performance.

In recent years, the impact of exercise training on autophagy becomes a hot spot in the field of exercise science. Exercise training can accelerate the metabolism of proteins, glucose, and fatty acids, improve mitochondrial biogenesis and promote angiogenesis, and delay the aging of skeletal muscle. These effects may be related to autophagy induced by exercise training. The reasonable mechanism of high altitude training-induced autophagy to improve exercise performance is summarized in Figure 1. High altitude training may induce autophagy and mitophagy so as to maintain the quality of skeletal muscle and remove dysfunctional mitochondria, thereby maintaining high efficiency of energy metabolism to meet the increased demand for energy. On the other hand, high altitude training may activate HIF-1 to stimulate the expression of EPO and VEGF, thereby increasing hemoglobin mass and capillary density of muscle. In conclusion, autophagy is a promoter for exercise performance during high altitude training. The underlying molecular mechanism of exercise-induced autophagy during hypoxic training is still unclear and less explored. Therefore, further studies are highly necessary. Hypoxic training-induced autophagy may provide a new field to explore the molecular mechanisms of adaptive response to high altitude training. Moreover, the exploration and validation of measurable autophagic biomarkers during high altitude training may have the potential for developing the strategies to monitor exercise fatigue, control training intensity, and conduct athlete talent screening.
